# ‘Repel all biters’: an enhanced collection of endophilic *Anopheles gambiae* and *Anopheles arabiensis* in CDC light-traps, from the Kagera Region of Tanzania, in the presence of a combination mosquito net impregnated with piperonyl butoxide and permethrin

**DOI:** 10.1186/s12936-017-1972-z

**Published:** 2017-08-15

**Authors:** Corey LeClair, Judith Cronery, Enock Kessy, Elsa V. E. Tomás, Yohannes Kulwa, Franklin W. Mosha, Mark Rowland, Natacha Protopopoff, J. Derek Charlwood

**Affiliations:** 1 Department of Disease Control, London School of Hygiene and Tropical Medicine, London, UK; 2 Kilimanjaro Christian Medical University College, Moshi, Tanzania; 3 MOZDAN, Morrumbene, Mozambique

**Keywords:** Light-trap, Mosquito, Sampling, Long lasting insecticidal nets, Resistance, Piperonyl butoxide, Surveillance, *Anopheles gambiae*, *Anopheles*

## Abstract

**Background:**

Mosquito nets containing synergists designed to overcome metabolic resistance mechanisms in vectors have been developed. These may enhance excitability in the mosquitoes and affect how they respond to CDC light-traps. Investigating the behaviour of vectors of disease in relation to novel mosquito nets is, therefore, essential for the design of sampling and surveillance systems.

**Methods:**

In an initial experiment in Muleba, Tanzania, nine bedrooms from three housing clusters were sampled. CDC light-traps were operated indoors next to occupied untreated nets (UTN), Olyset^®^ long lasting insecticidal net (LLIN) and Olyset Plus^®^ LLIN containing piperonyl butoxide (PBO) synergist. Nets were rotated daily between the nine rooms over nine nights. A further series of experiments using the nets on alternate nights in a single room was undertaken during the short rains. *Anopheles gambiae s.l.* were collected in CDC light-traps, a window-trap and Furvela tent-trap. *Anopheles gambiae s.l.* were identified to species by polymerase chain reaction (PCR).

**Results:**

In the initial experiment 97.7% of the 310 *An. gambiae s.l.* were *An. gambiae s.s.,* the remainder being *Anopheles arabiensis.* The number of mosquitoes collected from 81 light-trap collections was greater in the presence of an Olyset [density rate ratio 1.81, 95% CI (1.22–2.67), p = 0.003] relative to an UTN. In a second experiment, in the wet season 84% of the 180 *An. gambiae s.l.* identified were *An. arabiensis*. The number of *An. gambiae s.l.* collected from a light-trap compared to a tent-trap was significantly higher when an Olyset Plus net was used compared to an UTN. Survival of the mosquitoes in the window trap was not reduced by the use of an Olyset Plus net in the bedroom relative to an Olyset net.

**Conclusion:**

Mosquitoes entering bedrooms, even those susceptible to pyrethroids, were not killed by contact with an Olyset Plus LLIN. The enhanced numbers of *An. gambiae* or *An. arabiensis* collected in light-traps when a treated net is used requires further experimentation and may be because of a heightened escape reaction on the part of the mosquito.

## Background

Miniature CDC light-traps are commonly employed for surveillance of nocturnal, endophilic vectors of disease. The traps work by collecting unsuccessful host-seeking mosquitoes as they attempt to leave bedrooms in which the host is protected by a mosquito net [1–3]. Mosquito nets are required for monitoring anopheline malaria vectors since they are not attracted to light by itself and in the absence of a net they will feed and then rest. Wide-scale use of mosquito nets treated with insecticide (ITNs) often means that the householder is already sleeping under a treated net. This is often changed to an untreated nets (UTN) for such sampling at a considerable logistical inconvenience [4]. Changing the net is done because the pyrethroid component of the net is also a repellent, which may influence the efficiency of the light-trap. Previous work, conducted in Tanzania [5] and Zambia, comparing the numbers of *Anopheles arabiensis* collected in households with light-traps in the presence of deltamethrin-treated or UTNs, however, reported no significant difference in numbers caught [6]. Similarly, Kirby et al. [7]. found no difference in the numbers of *Anopheles gambiae s.l.* collected in The Gambia in light-traps hung within households in the presence of ITNs or UTNs. Resistant mosquitoes exhibit a reduced excito-repellency response to mosquito nets treated with pyrethroids [8–11]. It is possible that the mosquitoes in these studies were resistant to the insecticides and so were less affected by it than susceptible insects.

The two prominent resistance mechanisms are target-site resistance which reduces knock-down rates in mosquitoes and metabolic resistance in which there is a heightened enzyme activity so that the insecticide is metabolized and degraded before it can work [12]. The emergence of metabolic resistance has prompted the development of long-lasting insecticidal nets (LLINs) that incorporate synergists designed to overcome the enzymes responsible for resistance. The synergist, piperonyl butoxide (PBO), increases sensitivity to the insecticide by reducing the mosquito’s capacity to metabolize it. Increased sensitivity to the insecticide may affect the mosquito’s behaviour and affect how they respond to light-traps. The effect of different net types on mosquito catches was, therefore, examined from a village in northern Tanzania where a population of a pyrethroid-resistant *An. gambiae s.s.* is the primary vector.

## Methods

### Study site

The study took place in the village of Kakindo/Kyamyorwa B in Muleba District, Kagera Region in northwest Tanzania (02°04′27.5′′S, 31°34′10.8′′E). The village, located approximately 40 km outside of Muleba town and bisected by a two-lane highway connecting Bukoba and Mwanza, is separated by a floodplain, used for agricultural purposes, from an inlet of Lake Victoria. Houses are largely traditional, mud-walled, thatched-roofed structures although corrugated iron roofs are common and a number of houses are made of brick. The region has two rainy seasons: the main rains occur in March–May (average monthly rainfall 300 mm) and the secondary rains in October–December (average monthly rainfall 160 mm) [13]. Malaria is endemic with peaks of transmission at the end of the rainy seasons [13]. A pyrethroid-resistant population of *An. gambiae* is the primary vector in the area although *An. arabiensis* is also present [13, 14]. An initial study period took place between 7 and 16 July, 2014. The weather was dry and cool. Surface water was scarce, but due to a raised water table following the main rains, vegetation persisted and agricultural activities continued. The interior walls of houses in the village were sprayed with pirimiphos-methyl (Actellic 300 CS) in February and at the same time households in the village were provided with LLINs incorporating PBO (Olyset Plus) at the ratio of one net per two persons. A further series of experiments were undertaken during the short rains, between 3 December, 2015 and 12 January, 2016.

### Experimental design

In the initial study the effect of three different types of net on numbers of mosquito caught in light-traps run inside bedrooms was determined. The nets were: a standard 100-denier polyester UTN; a LLIN made from a high density (≥150 denier) polyethylene monofilament fibre incorporated with 2% w/w permethrin (Olyset^®^), and a recently developed LLIN (Olyset Plus^®^) similar in composition to the standard Olyset LLIN, but incorporating the synergist (10 g/kg) designed to reduce or neutralize any metabolic resistance conferred by mixed function oxidase mechanisms in *An. gambiae* [15]. The polymer composition of the two LLINs is different resulting in a different exposure of permethrin. Nine bedrooms, from three housing clusters were selected for the initial study. In each cluster, two bedrooms were separated by internal walls in a single house, and the third bedroom was located in a separate building. Two of the clusters were located on the floodplain side of the village and the third cluster was located in a more developed area of the village on the far side of the road. In each of the nine rooms, a CDC light-trap was hung 1.5 m from the floor close to the occupied mosquito net and operated from the time that the occupants went to bed to 06:30 the following day [16]. Three replicated Latin squares, with the three types of net rotated between the three rooms of each cluster, were undertaken. Thus, each net type was in use each night in every group, and three replicates were performed over nine collection nights. The number of adults and children differed between rooms but in each room it remained constant during the experiment.

In an attempt to refute data collected during the dry season, a further series of experiments were undertaken in a sentinel house used for routine sampling during the short rains. Prior to the experiment, in order to avoid possible bias due to any remaining effect of the pirimiphos-methyl, the walls of the house were plastered. A series of collections comprising a CDC light-trap, a window trap, and Furvela tent-trap [17] were conducted. Nets of the three types were used on alternate nights over a 41-day period. Prior to being used in the experiment the LLINs were suspended in the laboratory in Muleba to ensure that any excess insecticide had dispersed. The CDC light-trap and Furvela tent-trap were operated from 20:00 to dawn during each trapping evening. The Furvela tent-trap was a Nemo Losi 3 tent with a single occupant for all collections whilst two people (JDC and EVET) and their dog slept in the room with the light-trap.

Prior to the experiment, mosquitoes were only sampled from the window trap. Mosquitoes from three days of collection, when an Olyset Plus LLIN had been used in the bedroom, were provided with 10% glucose solution and kept in cups in the field laboratory. Survival at 24 and 48 h post-collection was determined. A knock-down bioassay using mosquitoes from the tent-trap collection was performed on five occasions. For these a 15-cm per side wire frame cube was converted into a cage by surrounding the frame with a mosquito net and closing the opening with a rubber band. Mosquitoes were held for an hour in the laboratory and then introduced into the cage. Non-flying mosquitoes had no alternative but to rest on the net. The number of mosquitoes knocked down each minute was determined by visual inspection of the floor of the cage. Numbers were converted into percentages of the total for subsequent analysis.

### Field processing

Collected mosquitoes were sexed, identified morphologically to genera or species [18, 19], and females classified into unfed, part-fed, fed, semi-gravid, and gravid groups, according to their abdominal condition [20] and counted. Female mosquitoes were stored individually over silica gel for subsequent processing. Specimens of the *An. gambiae* complex were later identified to species by multiplex real-time PCR TaqMan assay [21].

### Analysis

Household characteristics, entomological data and PCR species identification results were entered into a single database in Excel and analysed in Stata 12 [22]. Variables such as abdominal status, species, parity, net type and location were expressed as categorical variables.

For the main analysis, random-effects negative binomial regression was used to investigate the effect of different net types on numbers of *An. gambiae s.l.* collected in the light-traps and tent-traps. Since the nightly light-trap mosquito counts were overdispersed, negative binomial regression was used for the analysis, with adjustment for the effects of house cluster, persons per room, collection night and net type, and differences within these variables was expressed as density rate ratios (DRR) at the 95% significance level.

For the second experiment the number of *An. gambiae s.l*. captured in light-traps when different net types were used were compared using negative binomial regression to the numbers in the tent-trap (which acted as the control). The proportions of *An. gambiae s.l.* collected which were alive or dead, blood fed or unfed in the light-trap was calculated and compared using a Fisher’s Exact (two-tailed) test at the 95% significance level. The proportions of the total nightly catch (light-trap + window trap) sampled by window traps were calculated and compared for collections undertaken in the presence of an UTN or Olyset Plus LLIN using an unpaired two-sample *t* test.

### Ethics

The study was conducted as a component of the Pan African Malaria Vector Research Consortium project ‘Evaluation of a novel long-lasting insecticidal net and indoor residual spray product, separately and together, against malaria transmitted by pyrethroid resistant mosquitoes’ which received ethical clearance from the ethics review committees of the Kilimanjaro Christian Medical College (Certificate Number 781 on 16/09/2014), the Tanzanian National Institute for Medical Research (20/08/2014), and the London School of Hygiene and Tropical Medicine (reference 6551 on 24/07/2014). Prior to beginning collections, informal sensitization sessions were conducted with village members to explain sampling-related activities. Written informed consent was obtained from all participants who could withdraw from the study at any time should they wish to do so.

## Results

### Collection data

Three-hundred-and-three (97.7%) of the 310 *An. gambiae s.l.* specimens identified to species from the initial experiments were *An. gambiae* and the other seven were *An. arabiensis*. Therefore it is assumed that the unidentified samples at this time were also largely *An. gambiae* [23]. In the second experiment the species ratio had reversed. Of the 180 specimens identified to species at this time, 151 (84%) were *An. arabiensis.* For simplicity, it was also assumed that different members of the *An. gambiae* complex predominated during the two phases of the study. In the initial experiment, 395 *An. gambiae s.l.* were collected from the 81 light-trap nights; 98.4% of the *An. gambiae* collected were unfed (n = 389); of the remaining six, one was part-fed, four fed and one gravid. The *An. gambiae* population was in decline during the period of this experimental round. In addition to the *An gambiae s.l*., 26 *Anopheles funestus*, 243 *Coquillettidia fuscopennata* (a potential vector of Chikungunya and Sindbis viruses [24]), 24 *Mansonia* spp. and 800 other culicines, the majority of which were *Culex quinquefasciatus* were collected. Numbers of *An. gambiae s.l.* collected in the presence of an Olyset were significantly greater compared to an UTN (Table 1). There was no change in density ratios after adjustment for potential confounders such as date, location, persons per room and open or closed eaves. There was also no significant difference between numbers of *An. gambiae s.l.* captured with an Olyset compared to an Olyset Plus LLIN [DRR 0.79, 95% CI (0.55–1.12), p = 0.18]. There was no significant difference (p > 0.05) between the numbers of *C. fuscopennata* (Table 2) or other culicines (Table 3) collected by light-traps according to the net used. Similarly, there was no difference in the number of *C. fuscopennata* or other culicines collected when an Olyset or an Olyset Plus LLIN was used (p = 0.12, for both groups).

**Table 1:** Factors associated with the numbers of *Anopheles gambiae s.l.* captured during the dry season in light-traps, Kakindo, Tanzania

	Numbers collected^a^	Unadjusted		Adjusted model	
	Mean [95% CI], N	DRR [95% CI]	p-value	DRR [95% CI]	p-value
Intervention type					
Untreated Net	3.48 [2.21, 4.75], (94)	1.0		1.00	
Olyset Net	5.89 [3.87, 7.91], (159)	1.78	0.02	1.81 [1.22–2.67]	0.003
Olyset Plus Net	5.26 [3.43, 7.08], (142)	1.39	0.19	1.42 [0.94–2.14]	0.09

*CI* confidence interval

^a^Arithmetic mean number of *An. gambiae s.l.* trap/night

**Table 2:** Factors associated with the numbers of *Coquillettidia fuscopennata* captured during the dry season in light-traps, Kakindo, Tanzania

	Numbers collected	Unadjusted		Adjusted model	
	Mean^a^ [95% CI], (N)	DRR [95% CI]	p-value	DRR [95% CI]	p-value
Intervention type					
Untreated Net	3.07 [1.63, 4.52], (83)	1.00		1.00	
Olyset Net	3.59 [1.93, 5.26], (97)	0.95 [0.55–1.65]	0.64	1.12 [0.70–1.80]	0.63
Olyset Plus Net	2.33 [1.20, 3.46], (63)	0.69 [0.38–1.22]	0.42	0.75 [0.45–1.26]	0.28

*CI* confidence interval

^a^Arithmetic mean number of *Cq. fuscopennata* trap/night

**Table 3:** Factors associated with the numbers of *Culex* spp. captured in light-traps during the dry season, Kakindo, Tanzania

	Numbers collected	Unadjusted		Adjusted model	
	Mean^a^ [95% CI], (N)	DRR [95% CI]	p-value	DRR [95% CI]	p-value
Intervention type					
Untreated Net	9.93 [6.23, 13.62], (268)	1.00		1.00	
Olyset Net	11.89 [7.51, 16.27], (321)	1.08 [0.71–1.65]	0.50	1.13 [0.78–1.62]	0.52
Olyset Plus Net	7.82 [4.87, 10.76], (211)	0.67 [0.42–1.06]	0.38	0.73 [0.49–1.10]	0.14

*CI* confidence interval

^a^Arithmetic mean number of *Culex* spp. trap/night

During the sampling conducted during the short rains, the population of *An. arabiensis* was relatively stable. The proportion of engorged and part-fed female mosquitoes was significantly different when sampling was performed with an UTN relative to an Olyset or Olyset Plus [Fisher’s Exact test (two-tail) UTN/Olyset p < 0.001, UTN/Olyset Plus p < 0.001]. Despite the provision of sugar solution and usage of a conical collection bag, only 10% of the mosquitoes were alive in the morning. The proportion of *An. arabiensis* collected alive or dead was not affected by the type of net in use [Fishers Exact (two tail) UTN/Olyset p = 0.9, UTN/Olyset Plus p = 1.0, Olyset/Olyset Plus p = 0.9]. The total nightly indoor collection (light-trap and window-trap), which was always small compared to the light-trap, was also independent of the net in use.

In the second experiment, in the presence of an Olyset Plus, significantly more *An. arabiensis* were collected in the light-trap compared to the tent-trap [DRR 2.08, 95% CI (2.30–3.32), p = 0.002] whereas the numbers collected when an Olyset LLIN or UTN was present were not significantly different [DRR 0.9, 95% CI (0.45–1.79) p = 0.76] and [DRR 1.41, 95% CI (0.63–3.16), p = 0.4], respectively (Table 4). The numbers of *An. arabiensis* collected in tent-traps did not appear to be influenced by the type of net in use indoors (Table 5). The percentage of insects that had succeeded in obtaining a blood meal decreased from 23.7% (98 of 413 examined) with an UTN, to 12.9% (36 of 279 examined) with the standard Olyset to 8.2% (179 of 2184 examined) with the Olyset Plus.

**Table 4:** *Anopheles gambiae s.l.* captured during the wet season in the presence of three different bednet types, Kakindo, Tanzania

Net type	Collection (N)	Light-trap Mean^a^ [95% CI]	Tent-trap Mean^a^ [95% CI]	Light-trap: Tent-trap DRR [95% CI]	p-value
Untreated	5	70.1, [31.4–156.2]	78.4, [25.7–239.0]	0.9, [0.45–1.79]	0.76
Olyset	3	87.5, [29.0–264.5]	53.3, [6.70–422.6]	1.41, [0.63–3.16]	0.4
Olyset Plus	13	161.4, [134.7–193.4]	72.6, [58.6–90.0]	2.08, [2.30–3.32]	0.002

^a^Arithmetic mean number of *An. gambiae s.l* trap/night

**Table 5:** *Anopheles gambiae s.l.* captured during the wet season in the presence of three different bednet types, Kakindo, Tanzania

Net type	Light-trap collection (N)	Light-trap DRR [95% CI]	p-value	Tent-trap collection (N)	Tent-trap DRR [95% CI]	p-value
Untreated	5	1.0	–	8	1.00	–
Olyset	3	1.13, [0.56, 2.28]	0.74	5	0.58, [0.32, 1.06]	0.08
Olyset Plus	13	1.81, [1.11, 2.96]	0.02	9	0.83, [0.50, 1.37]	0.46

All sampled mosquitoes from the window trap were alive at the time of collection in the morning. The non-removal of a sample of dead insects in placed in the trap, and the absence of ants, indicates that both live and dead mosquitoes would have been seen if they had been there. All 125 mosquitoes from three days of collection, when an Olyset Plus had been used in the bedroom, were alive at 24 and 48 h post-collection.

**Fig. 1:**
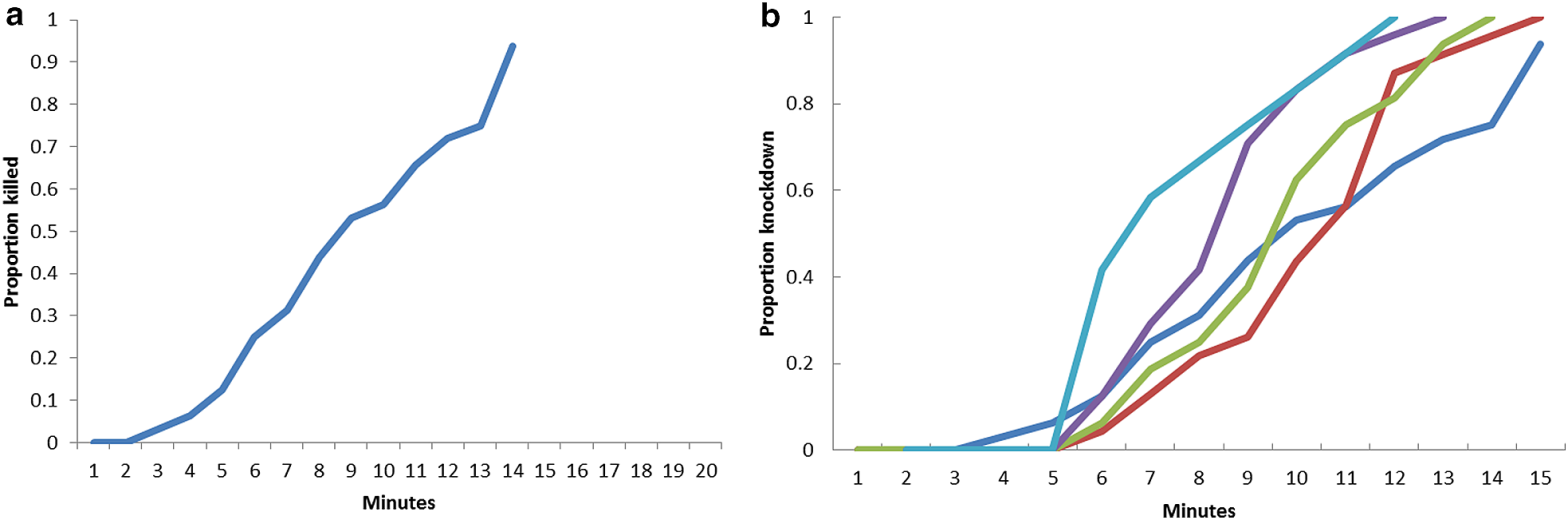
Proportion of *Anopheles gambiae s.l.* (84% *Anopheles arabiensis)* captured in tent-traps during the wet season killed and knocked down when exposed to Olyset Net. **a** Proportion of *An. gambiae s.l. (*84% *An.* arabiensis) killed per minute upon exposure to an Olyset net bioassay. **b** Proportion of *An. gambiae s.l.* (84% *An. arabiensis*) knocked down per minute upon exposure to an Olyset net bioassay. *Colours indicating individual bioassays completed

The standard Olyset by itself knocked down (and killed) *An. arabiensis* within 15 min of exposure in the initial bioassay when all three net types were tested simultaneously. Given the absence of mortality in the cage made of untreated netting, and that the mortality seen in the Olyset Plus cage was the same as that observed in the standard Olyset, further bioassays were concentrated on the standard Olyset alone (Fig. 1). In these, knock-down started approximately 3 min after exposure and by 15 min all insects in all replicates were knocked down. Further examination of these insects indicated that they were dead and implies that *An. arabiensis* remained susceptible to the insecticide even without the addition of PBO.

## Discussion

The results obtained from both sets of experiments indicate that the type of mosquito net in use affected the numbers of mosquitoes caught by light-traps. Surprisingly, significantly fewer mosquitoes, both resistant *An. gambiae* in the dry season and susceptible *An. arabiensis* in the wet season, were caught when an UTN was used compared to the standard Olyset or Olyset Plus. If, as seems reasonable based on previous hut trials in Benin [25], the net in use did not affect the number of *An. gambiae s.l.* entering houses, deterrent effect [26], and the traps functioned correctly and since (in the second experiment at least) the hosts did not differ between nights, then the results indicate that the insects’ behaviour was affected by encountering the net. How or why remains unknown. One possible explanation is that, given the differences in polymer composition between the two treated nets, surface exposed containing permethrin and evaporation rates are likely to differ which may affect behaviour. Similarly, the PBO on the Olyset Plus, by reducing the insects’ ability to metabolize the insecticide it would have affected the irritability of the insect without, or before, having a killing effect. This irritability may induce a more direct escape reaction on the part of the insect compared to one that has been unable to feed through an UTN, which may make a more indirect and less ‘driven’ exit. An irritated insect may, therefore, be more likely to fly towards the light emitted from a light-trap than a non-irritated one. Insects in the latter category may leave through other openings in the room with fewer going to the light-trap or window-trap as demonstrated by the light-trap/window-trap ratio when an UTN is used (Table 6).

**Table 6:** *Anopheles gambiae s.l.* captured indoors during the wet season in the presence of three different bednet types, Kakindo, Tanzania

Net type	Collection (N)	Light-trap Mean^a^ [95% CI]	Window-trap Mean^b^ [95% CI]	Light/window Mean ratio [95% CI]
Untreated	5	70.1, [31.4–156.2]	2.4, [1.1–5.3]	29.2, [11.4–74.4]
Olyset	3	87.5, [29.0–264.5]	–	–
Olyset Plus	13	161.4, [134.7–193.4]	2.7, [1.6–4.4]	63.4, [38.5–104.4]

^a^Geometric mean number of *An. gambiae s.l.* captured

^b^Geometric mean calculated from 12 collections with Olyset Plus

The results of the bioassay conducted with the standard Olyset in the wet season indicated that the *An. arabiensis* population was susceptible to pyrethroids [27]. On the other hand, the population of *An. gambiae* in Muleba is highly resistant to this group of insecticides [13, 14, 27]. Nevertheless, independent of resistance status, mosquitoes entering bedrooms were not killed by contact with an Olyset Plus as shown by 100% survival of the mosquitoes collected from window-traps. Mosquitoes were, however, prevented from successfully obtaining a blood meal on a nearby, unprotected host (the dog).

## Conclusion

In many areas people may possess LLINs but may not use them. Non-LLIN-using households may be at a greater risk when PBO-containing nets are used by their neighbours, as they may be when topical repellents are used [28]. Distribution of LLINs containing PBO should be accompanied by public health messaging to ensure high LLIN utilization in order to avoid the potential of enhanced risk of infection by those persons not using them.
